# Transcriptional dysregulation in the cerebellum triggered by oligodendroglial α-synucleinopathy: insights from a transgenic mouse into the early disease mechanisms of MSA

**DOI:** 10.1007/s00702-025-02892-5

**Published:** 2025-02-15

**Authors:** Antonio Heras-Garvin, Lisa Fellner, Roberta Granata, Gregor K. Wenning, Nadia Stefanova

**Affiliations:** Laboratory for Translational Neurodegeneration Research, Division of Neurobiology, Department of Neurology, Medical University of Innsbruck, Innrain 66, 3rd floor, Innsbruck, 6020 Austria

**Keywords:** Multiple system atrophy, Α-synucleinopathy, RNA-seq, Cerebellum, Neurodegeneration, Aging

## Abstract

Multiple system atrophy (MSA) is a fatal neurodegenerative disorder characterized by abnormal accumulation of α-synuclein, progressive neuronal loss, motor impairment and widespread pathological changes, which include significant involvement of the cerebellum. To understand the early molecular mechanisms that might underlie α-synuclein-triggered MSA cerebellar pathology, we performed RNA sequencing (RNA-Seq) of cerebellar samples from a well-established model of MSA. RNA-Seq and differential gene expression analysis was conducted in the PLP-αSyn model of MSA. Cerebellum from two and 12-month-old MSA and wildtype mice were used. Gene ontology (GO) and KEGG enrichment analyses of the differentially expressed genes (DEGs) were performed to explore processes involved in MSA-like disease progression. The overlap between transcriptional changes in MSA and those associated with aging was also evaluated. RNA-Seq analysis demonstrated significant transcriptional dysregulation in cerebellum from MSA mice, even at early stages. GO and KEGG analyses of DEGs point to a potential role of synaptic dysfunction, cellular signaling dysregulation and inflammation in the cerebellar pathology of MSA mice. In addition, those changes exacerbate with disease progression. Additionally, our analysis of aging in both control and PLP-αSyn mice showed that age-related transcriptional changes in mid-aged controls seem to be present in young MSA mice. Thus, MSA-like pathology might lead to an acceleration of aging-related mechanisms. Our findings demonstrate significant cerebellar transcriptional dysregulation triggered by oligodendroglial α-synucleinopathy in PLP-αSyn mice, revealing pathways that might be critical for the early cerebellar pathology of MSA, and that may serve as potential molecular targets for therapeutic interventions in this devastating disorder.

**Supplementary Information:**

The online version contains supplementary material available at 10.1007/s00702-025-02892-5.

## Introduction

Multiple system atrophy (MSA) is a relentlessly progressive atypical parkinsonian disorder leading to severe motor disability and death few years after symptom-onset with no treatment available to mitigate symptom severity or clinical progression. The prevalence of MSA is estimated at 4/100,000 people, qualifying MSA as an orphan disease accounting for over 30,000 sufferers in the European Union (EU) (Stefanova et al. 2009; Wenning et al. 2013). MSA patients usually develop different autonomic cardiovascular and respiratory problems in a premotor phase of the disease that can last for months to years, followed by the appearance of motor symptoms that worsen through the disease course (Fanciulli and Wenning 2015). MSA belongs to a group of neurodegenerative disorders, the α-synucleinopathies, which are characterized by the abnormal accumulation of α-synuclein (α-syn). In contrast to Parkinson’s disease (PD) and Dementia with Lewy bodies (DLB), where α-syn mainly accumulates in neurons in Lewy bodies, in MSA α-syn mainly accumulates in oligodendroglial cells in form of glial cytoplasmic inclusions (GCIs), the histopathological hallmark of MSA (Fanciulli and Wenning 2015; Spillantini et al. 1997; Trojanowski and Lee 2003; Tu et al. 1998). α-syn accumulation in MSA leads to glial and neuronal dysfunction, neuroinflammation and finally neurodegeneration (Fanciulli and Wenning 2015). Based on its clinical presentation, MSA is subdivided in two main variants, the parkinsonian (MSA-P) and cerebellar (MSA-C), which usually overlap at different degrees during the disease progression (Fanciulli and Wenning 2015). Postmortem neuropathological analysis defines the final diagnosis of MSA by proving α-syn-positive oligodendroglial inclusions and selective neurodegeneration. In MSA-P, striatonigral degeneration (SND), involving the putamen, globus pallidus, and substantia nigra, leads to parkinsonian symptoms such as rigidity and bradykinesia. In MSA-C, the cerebellum, pons, and inferior olivary nuclei are primarily affected, causing ataxia and coordination problems. Based on the evidence obtained from preclinical models and postmortem analyses of patients, it has been suggested that MSA could be considered primarily as an oligodendrogliopathy, however the mechanisms underlying its pathogenesis and the sequence of molecular events behind disease progression have been only partially deciphered (Ahmed et al. 2012; Fanciulli and Wenning 2015; Wenning et al. 2008).

In recent years, increasing evidence supports the prion-like behavior of α-syn due to its ability to induce the aggregation of native α-syn into fibrillary structures and pathogenic inclusions (Hijaz and Volpicelli-Daley 2020). In addition, several studies have shown the existence of different α-syn strains or conformations (Heras-Garvin and Stefanova 2020). MSA-derived α-syn seems to possess higher infectivity and pathogenic potential than PD or DLB-derived α-syn, which might be explained by biochemical and structural differences between these α-syn strains (Bernis et al. 2015; Holec and Woerman 2021; Schweighauser et al. 2020; Shahnawaz et al. 2020; Tarutani et al. 2018; Van der Perren et al. 2020; Yamasaki et al. 2019). The fact that MSA-derived α-syn induces a more significant motor impairment, neurodegeneration, neuroinflammation and α-syn seeding and spreading than PD and DLB strains in experimental models could explain the more aggressive nature of this disease compared to other α-synucleinopathies (Van der Perren et al. 2020). What causes the generation of different strains and their properties remains poorly understood. However, it has been proposed that different intracellular milieus might generate distinct α-syn strains (Peng et al. 2018). Experimental data indicates that α-syn strains formed in the cellular environment of oligodendrocytes obtain a more compact structure and achieve higher potency in seeding aggregation with enhanced neurodegenerative potential, leading to a more aggressive disease course (Ferreira et al. 2021; Peng et al. 2018). At the cellular level, α-syn can be found in different cellular organelles, including the nucleus, where it functions as a regulator of gene expression through its direct interaction with DNA (Pinho et al. 2019). Thus, we hypothesized that the oligodendroglial α-synucleinopathy observed in MSA could be associated with important region-specific transcriptional changes that may precede the neurodegenerative process and play a central role on disease progression.

The PLP-αSyn transgenic mouse model recapitulates many of the clinical (motor and non-motor) and pathological features of MSA (Boudes et al. 2013; Krismer et al. 2013; Kuzdas et al. 2013; Refolo et al. 2018; Stefanova et al. 2005, 2007; Stemberger et al. 2010). Generated in a C57BL/6 N background, these animals overexpress human wildtype α-syn specifically in oligodendrocytes under the myelin proteolipid protein (PLP) promoter, which leads to α-syn accumulation and the formation of GCI-like inclusions in the cytoplasm of oligodendrocytes (Kahle et al. 2002). PLP-αSyn mice present a premotor phase of the disease with autonomic and REM-sleep disorder symptoms that is already present at 2–3 months of age, followed by the progressive loss of dopaminergic neurons in the substantia nigra pars compacta at 6-month-old and the loss of dopaminergic terminals and medium spiny neurons in the striatum at 12-month-old (Bassil et al. 2016; Refolo et al. 2018). SND in these mice is linked to a significant and progressive motor impairment (Refolo et al. 2018; Stefanova et al. 2005) and region-specific microglial activation (Refolo et al. 2018; Stefanova et al. 2007). Interestingly, the cerebellum and its associated circuitry, which are crucial in MSA-C, are not as profoundly affected in PLP-αSyn mice up to 12 months of age. These mice do not show clear degeneration of Purkinje cells, pontocerebellar pathways and associated structures at the time of significant nigral and striatal degeneration. However, secondary insults such as proteasome impairment or 3-nitropropionic acid (3-NP) injection can exacerbate or induce cerebellar pathology in this transgenic mouse model, including the loss of Purkinje cells and olivopontocerebellar degeneration (Stefanova et al. 2005, 2012). These latter observations suggest the existence of subtle or latent changes in the cerebellum of PLP-αSyn mice associated with the oligodendroglial α-synucleinopathy even under baseline conditions, which are then amplified or accelerated by a secondary insult.

Thus, the PLP-αSyn mouse model provides an important and relevant pre-clinical tool to study the sequence of molecular events underlying neurodegeneration in oligodendroglial α-synucleinopathy, including region-specific transcriptional changes that might contribute to disease progression. In this regard, two previous studies by our group demonstrated gene dysregulation in the brain of old MSA mice (Nicholson et al. 2024) and the substantia nigra of PLP-αSyn mice in the very early, non-symptomatic stage of the disease (Schafferer et al. 2016). Here, we analysed the transcriptional differences between the cerebellum of MSA mice and wildtype controls throughout disease progression.

## Methods

### Animals

PLP-αSyn mice (MGI:3604008) and C57BL/6 N wildtype (WT) animals were kept under temperature-controlled pathogen-free conditions with a light/dark 12 h cycle. Two-month-old and twelve-month-old PLP-α-syn and WT mice were used in this study, 8 mice per group with both sexes equally represented. All experiments were performed according to the ethical guidelines with the permission of the Austrian Federal Ministry of Science and Research (BMFWF-2022-0.899.398).

### RNA processing and sequencing

Mice were perfused intracardially with phosphate-buffered saline (PBS, pH 7.4, Sigma) under deep thiopental anaesthesia. Brains were quickly extracted, and the entire cerebellum from each animal was dissected on ice and frozen in liquid nitrogen. Total RNA was extracted using the standard TRIzol method (Thermo Fisher) followed by isolation in RNeasy Midi Spin Columns (Qiagen), treated with RNase-free DNase (Qiagen), and dissolved in DEPC-water. RNA quality was analysed using an Agilent Tapestation. PolyA-enriched libraries were generated by Novogene UK and sequenced at Illumina Novaseq 6000 platform at 150 bp PE, generating on average 70 million reads per sample.

RNA-seq raw reads were trimmed for residual adapter sequences and low quality sequences were removed using cutadapt (Martin 2011). RNA-seq reads were mapped to mouse and human reference genome (mm10 and hg38 respectively) with STAR aligner (Dobin et al. 2013). Read counts were obtained using featureCounts (Liao et al. 2019) and normalized using the normalization algorithms of DESeq2 (Love et al. 2014). Differential gene expression analysis of control versus age-matched PLP-αSyn mice or between young vs. old mice were performed in DESeq2 accounting for hidden batch effects by the removal of unwanted variation (RUVg, k3) method (Risso et al. 2014). A threshold cut‐off of adjusted p-value < 0.05 was applied (Benjamini-Hochberg procedure, which controls false discovery rate (FDR).

### Gene ontology and KEGG enrichment analysis

Gene ontology (GO) and KEGG enrichment analysis of differentially expressed genes was performed with Metascape (Zhou et al. 2019) to identify overrepresented GO and KEGG terms in the cerebellum affected by genotype and/or aging. All annotated genes in the genome were employed as a background gene list. GO Biological Processes, GO Cellular components were used as ontology sources. GO and KEGG terms with a p value < 0.01, a minimum count of 3, and an enrichment factor > 1.5 were filtered and collected. P values were calculated based on a cumulative hypergeometric distribution, and q-values were calculated using the Benjamini-Hochberg FDR procedure for multiple testing. KEGG plots were generated with SRplot (Tang et al. 2023).

## Results

### Significant transcriptional changes in the cerebellum of MSA mice are evident at early disease stages

**Fig. 1:**
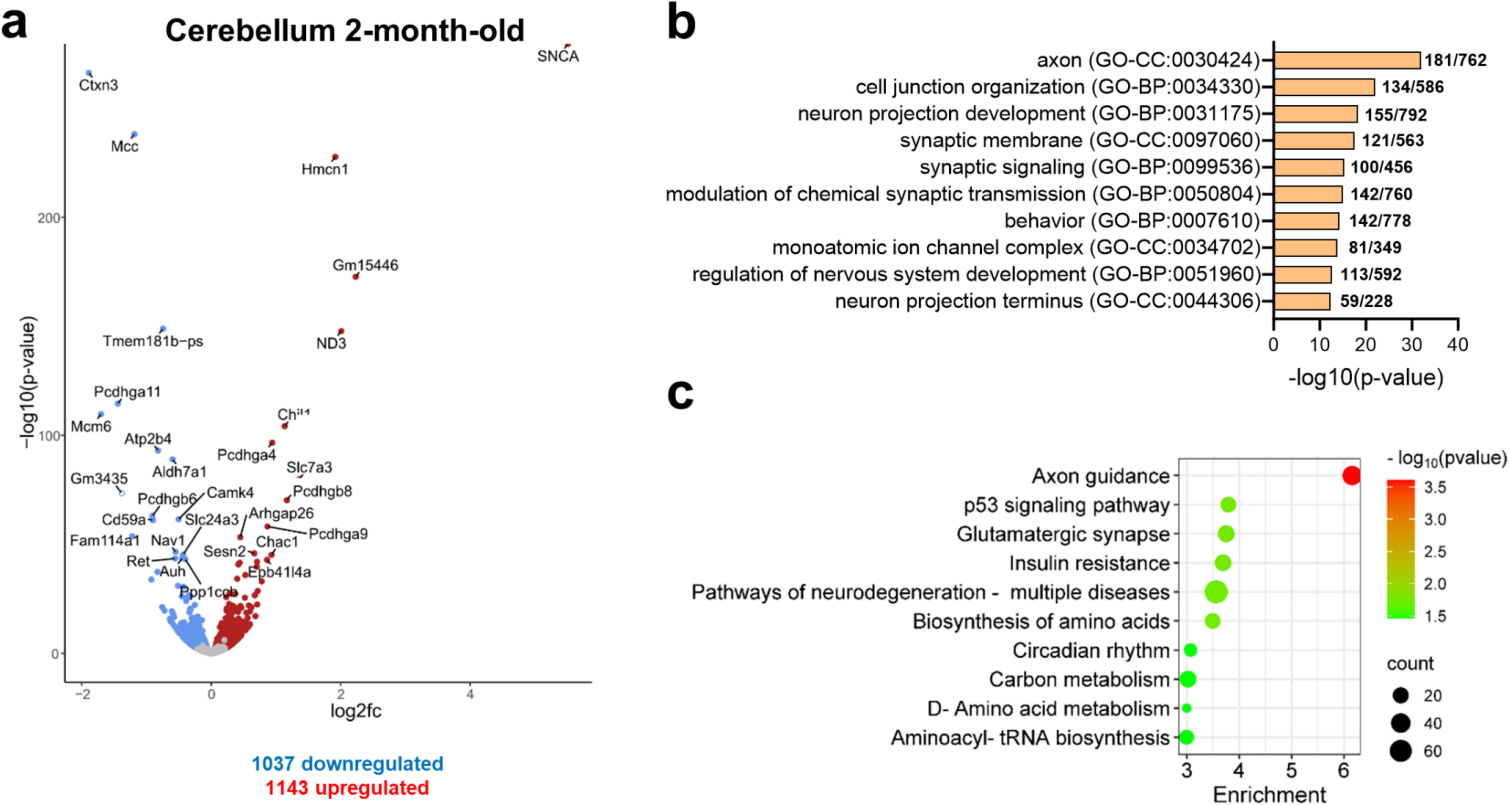
RNA-Seq analysis of cerebellum of 2-month-old PLP-αSyn mice versus wildtype controls. **A**, Volcano plot showing the differentially expressed genes (DEGs) in cerebellum of MSA vs. control mice. Blue: downregulated; Red: upregulated; Grey: non-significant. DEGs with an adjusted p-value < 0.05 were considered significant. The top 30 DEGs appear labelled. Log2fc: Log2 Fold-change. **B**, Top 10 GO clusters with their representative enriched terms (one per cluster) enriched in cerebellum of MSA mice. GO-BP: biological processes; GO-CC: cellular components; GO-MF: molecular function. The graph also includes the number of DEGs associated with each GO term relative to the total number of genes described to be associated with that term. **C**, Top10 KEGG pathways enriched in cerebellum of MSA mice. Counts represent the number of DEGs from the analysis that is associated with each KEGG pathway

DESeq2 analysis of cerebellar tissue from 2-month-old PLP-αSyn and wildtype (WT) control mice identified a significant number of differentially expressed genes (DEGs) in the cerebellum of MSA mice (Fig. 1a and Supplementary file). As expected, the most upregulated gene in the cerebella of PLP-αSyn mice was human synuclein (*SNCA*), which is overexpressed in these transgenic mice. Among the top 30 dysregulated genes, we identified genes involved in neuronal and synaptic function (*Ctxn3*, *Mcc*, *Nav1*, *Pcdhga11*, *Pcdhga4*, *Pcdhgb8*, *Pcdhgb6*, *Pcdhga9*, *Ppp1ccb*, *Ret*, *Slc7a3*), neuroinflammation (*Arhgap26*, *Cd59a*, *Chil1*, *Fam114a1*, *Ppp1ccb*), oxidative stress response (*Aldh7a1*, *Auh*, *Chac1*, *Sesn2*), calcium signalling (*Atp2b4*, *Camk4*), mitochondrial function and energy metabolism (*Auh*, *ND3*, *Slc24a3*). GO analysis of all significant DEGs in young MSA mice identified key biological processes and cellular components that could be associated with cerebellar dysfunction (Fig. 1b). The enrichment of terms like “axon”, “neuron projection development” and “cell junction organization” suggest impaired axonal structure and neuronal connectivity. In addition, the terms such as “synaptic membrane”, “synaptic signaling” and “modulation of chemical synaptic transmission” highlights deficits in synaptic communication, while “monoatomic ion channel complex” could be associated with ion channel dysregulation, which may affect neuronal excitability. Functional and developmental impairments are further suggested by terms like “behavior” and “regulation of nervous system development”. Similarly, KEGG pathway analysis indicated enrichment in various pathways, with the “axon guidance” pathway being particularly prominent (Fig. 1c). These findings imply that neuronal connectivity and synaptic function may be impaired or disrupted in the cerebellum of MSA mice with oligodendroglial α-synucleinopathy, even at early disease stages.

### Transcriptional changes in the cerebellum of MSA mice persist and/or aggravate through disease progression

**Fig. 2:**
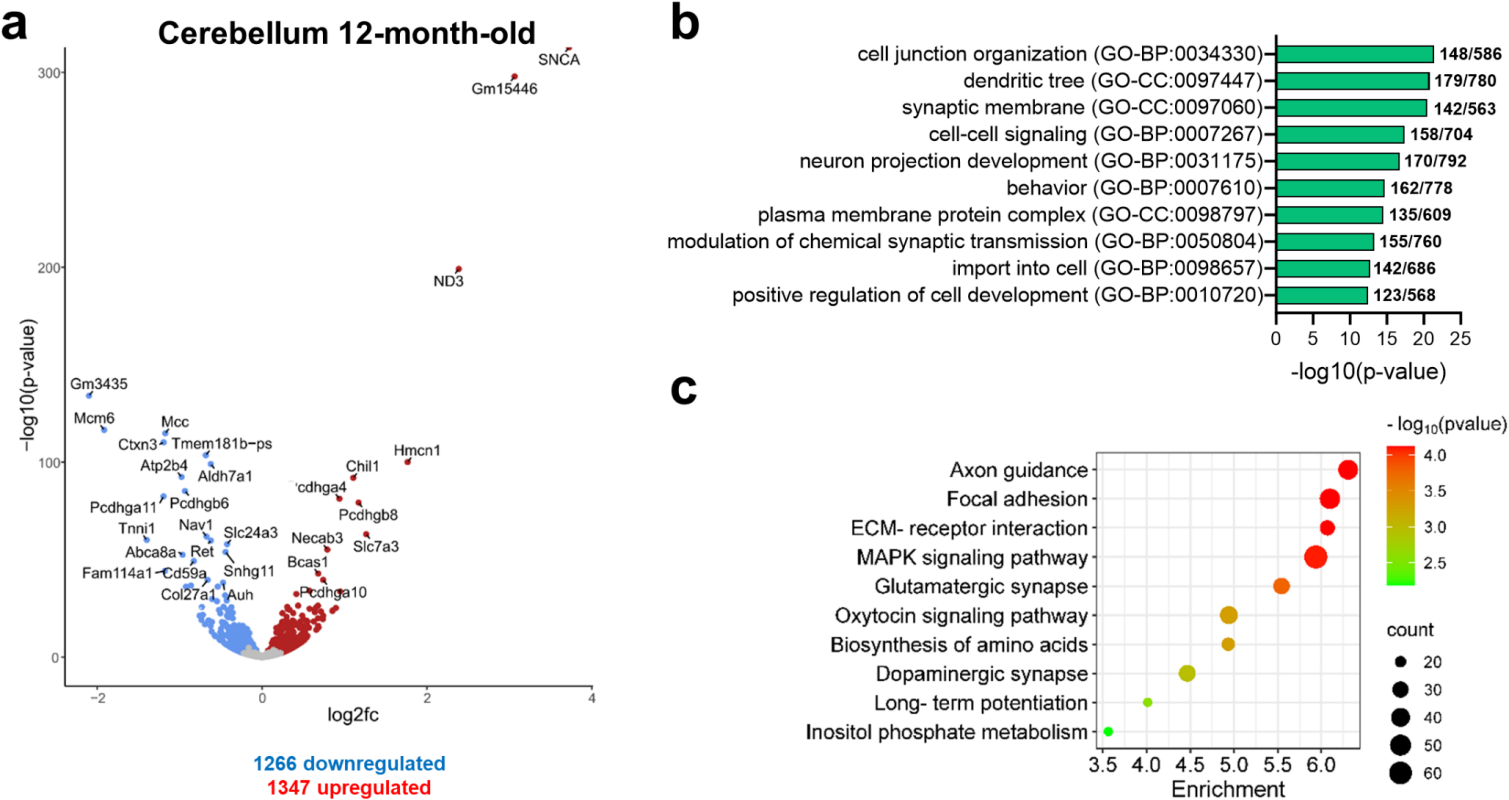
RNA-Seq analysis of cerebellum of 12-month-old PLP-αSyn mice versus wildtype controls. **A**, Volcano plot showing the DEGs in cerebellum of MSA vs. control mice. Blue: downregulated; Red: upregulated; Grey: non-significant. DEGs with an adjusted p-value < 0.05 were considered significant. The top 30 DEGs appear labelled. Log2fc: Log2 Fold-change. **B**, Top 10 GO clusters enriched in cerebellum of old MSA mice. **C**, Top10 KEGG pathways enriched in cerebellum of old MSA mice. Counts represent the number of DEGs from the analysis that is associated with each KEGG pathway

At 12 months of age, DESeq2 analysis of cerebellar samples revealed a higher number of DEGs between MSA and control mice compared to 2-month-old animals (Fig. 2a and Supplementary file). However, the top DEGs in older PLP-αSyn mice were similar to those in younger mice, with a prominent and significant upregulation of SNCA, followed by genes associated with axon guidance, neuronal function, synaptic activity (*Ctxn3*, *Hmcn1*, *Mcc*, *Nav1*, *Necab3*, *Pcdhgb6*, *Pcdhga11*, *Pcdhga4*, *Pcdhgb8*, *Pcdhga10*), calcium signalling and homeostasis (*Atp2b4* and *Slc24a3*), oxidative stress and metabolic processes (*Aldh7a1*, *Auh* and *ND3*) and neuroinflammation and immune response (*Cd59a*, *Chil1*, *Fam114a1*). In addition, we also found dysregulated genes involved in cytoskeletal and structural integrity (*Col27a1*, *Tnni1*), regulation of non-coding RNA (*Snhg11*) and lipid metabolism and transport (*Abca8a*, *Bcas1*). Interestingly, *Abca8a* plays a role in lipid transport and homeostasis, which is essential for maintaining myelin and neuronal membrane integrity (Kim et al. 2013) and has been linked to human MSA pathogenesis (Bleasel et al. 2013), while *Bcas1* is linked to oligodendrocyte maturation and myelin formation (Ishimoto et al. 2017; Liu et al. 2022). Thus, the dysregulation of these genes suggests myelination impairment in aged MSA mice.

GO enrichment analysis of significant DEGs in the cerebellum of 12-month-old MSA mice revealed an enrichment of GO terms similar to those observed in younger mice, primarily associated with disruption in neuronal connectivity, dendritic architecture, impaired neurotransmitter dynamics, and synaptic structure (Fig. 2b). As in 2-month-old mice, enrichment in “behaviour” suggests a potential link to motor and cognitive dysfunction. Additionally, “positive regulation of cell development” and “import into cell” terms may reflect compensatory or stress-related mechanisms within cerebellar circuits, such as cellular growth or differentiation, phagocytosis and autophagy. KEGG analysis of DEGs in 12-month-old MSA mice showed a significant enrichment in pathways associated with neuronal connectivity, synaptic function, and cellular signalling (Fig. 2c). The enrichment of the “axon guidance” and “glutamatergic synapse” pathways were significantly greater in PLP-αSyn mice at 12 months compared to 2 months, suggesting a more advanced disruption in neuronal connectivity and synaptic function with disease progression. Furthermore, the “dopaminergic synapse” pathway was also enriched at 12 months, indicating alterations in dopaminergic signalling. In addition, enrichment of the “MAPK signaling” pathway also points to the activation of stress response and cellular signalling mechanisms. These findings support a progressive neurodegenerative process in the cerebellum of MSA mice associated with the oligodendroglial α-synucleinopathy.

**Fig. 3:**
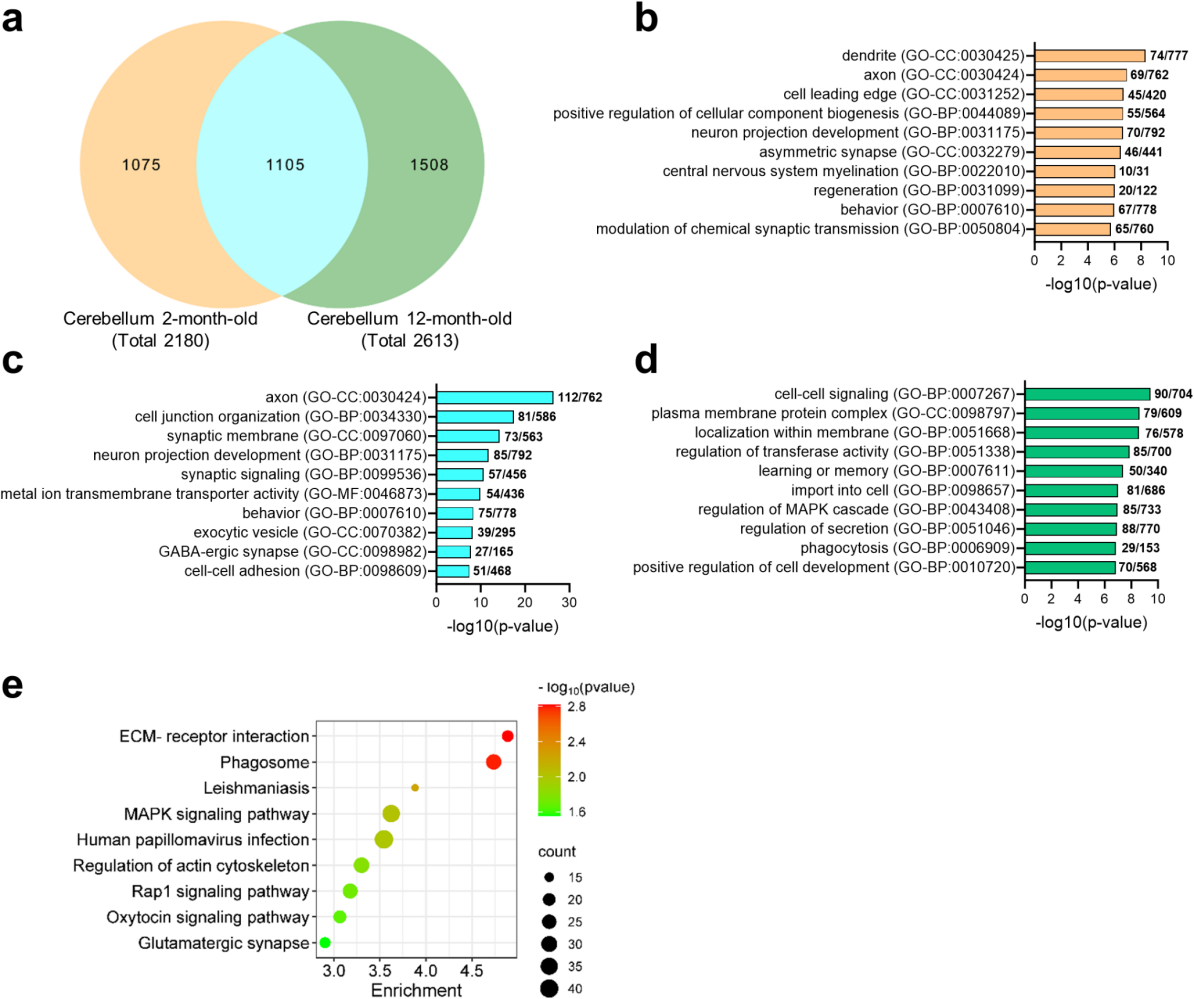
Shared and specific DEGs in the cerebellum of MSA mice at the two disease stages. **A**, Venn Diagram indicating the overlap between DEGs at 2 months and 12 months in PLP-αSyn mice. **B**, Top 10 GO clusters from genes specifically dysregulated in two-month-old MSA mice. **C**, Top 10 GO clusters from genes dysregulated in both young and old MSA mice. **D**, Top 10 GO clusters from genes specifically dysregulated in 12-month-old MSA mice. **E**, KEGG pathways from DEGs specifically in old MSA mice. KEGG analysis of shared DEGs or specifically dysregulated in young MSA mice did not show significant pathway enrichment

A comparison between the DEGs in the cerebellum of MSA mice at 2 and 12 months demonstrated the existence of an important number of genes that were dysregulated at both disease stages, but also of genes that were specifically dysregulated at 2 or 12 months respectively (Fig. 3a). GO analysis of genes specifically dysregulated in 2-month-old MSA mice indicate early disruptions in neuronal and synaptic structures (Fig. 3b). Enrichment of terms “dendrite”, “axon”, and “asymmetric synapse” suggest impairments in neural connectivity and communication. The enrichment of “central nervous system myelination” point towards early dysregulation of myelination, while terms like “regeneration” and “positive regulation of cellular component biogenesis” suggest compensatory responses, reflecting a potential attempt to counteract structural damage. Shared DEGs between two and 12-month-old PLP-αSyn mice emphasize persistent deficits in neuronal connectivity and synaptic organization, as suggested by terms like “axon”, “synaptic membrane”, “cell junction organization”, “neuronal projection development” and “synaptic function” (Fig. 3c). Processes related to “synaptic signaling”, “GABAergic synapse” and “cell-cell adhesion” suggest enduring imbalances in neurotransmission that could be essential for motor and cognitive functions. In this regard, the consistent enrichment in “behaviour” supports the functional consequences of these molecular changes. At 12 months, GO analysis of the specific DEGs demonstrated the emergence of new processes (Fig. 3d), including “learning or memory”, “regulation of MAPK cascade” and “phagocytosis”, pointing to advance-stage responses such as cellular stress, neuroinflammation, and compensatory mechanisms. Enrichment in “cell-cell signaling” and “localization within membrane” may reflect growing disruptions in intercellular communication and membrane dynamics. Additionally, terms like “import into cell” and “regulation of secretion” indicate altered intracellular trafficking and vesicle dynamics, likely contributing to disease progression. KEGG analysis of specific DEGs in 12-month-old MSA mice revealed significant enrichment in several biological pathways, highlighting the involvement of key pathological processes in the advanced stages of the disease (Fig. 3e). Pathways such as “focal adhesion”, “ECM-receptor interaction” and “phagosome” suggest alterations in immune responses, cellular adhesion, and extracellular matrix (ECM) remodelling, which may contribute to neurodegenerative progression. Additionally, the “Leishmaniasis”, “MAPK signaling”, “human papillomavirus infection” and “Rap1 signaling” pathways indicate disruptions in cellular signalling, stress response mechanisms, and immune-related processes, providing evidence of escalating inflammation as the disease progresses. Enrichment in the “glutamatergic synapse” pathway further suggests synaptic dysfunction. The “regulation of actin cytoskeleton” pathway also points to cytoskeletal remodelling, which may affect neuronal morphology and synaptic integrity, contributing to disease progression. In contrast, KEGG analysis of specific DEGs at 2 months revealed no significant pathway enrichment, nor did the analysis of shared DEGs at 2 and 12 months, indicating that the early molecular alterations in MSA might be relatively less prominent or in an early phase.

Overall, these findings highlight immune dysregulation, cellular stress, and synaptic dysfunction as key features of MSA progression. In the early stages, disruptions in synaptic function, neuronal connectivity and myelination are observed. Throughout the disease, pathways related to synaptic dysfunction, cellular signalling, and immune responses remain involved. However, at late stages, the disease shows significant enrichment in pathways linked to immune dysregulation, cellular stress, inflammation, and alterations in extracellular matrix remodelling, supporting an escalating role of neuroinflammation as the disease progresses.

### Oligodendroglial α-synucleinopathy exacerbates age-related transcriptional changes

**Fig. 4:**
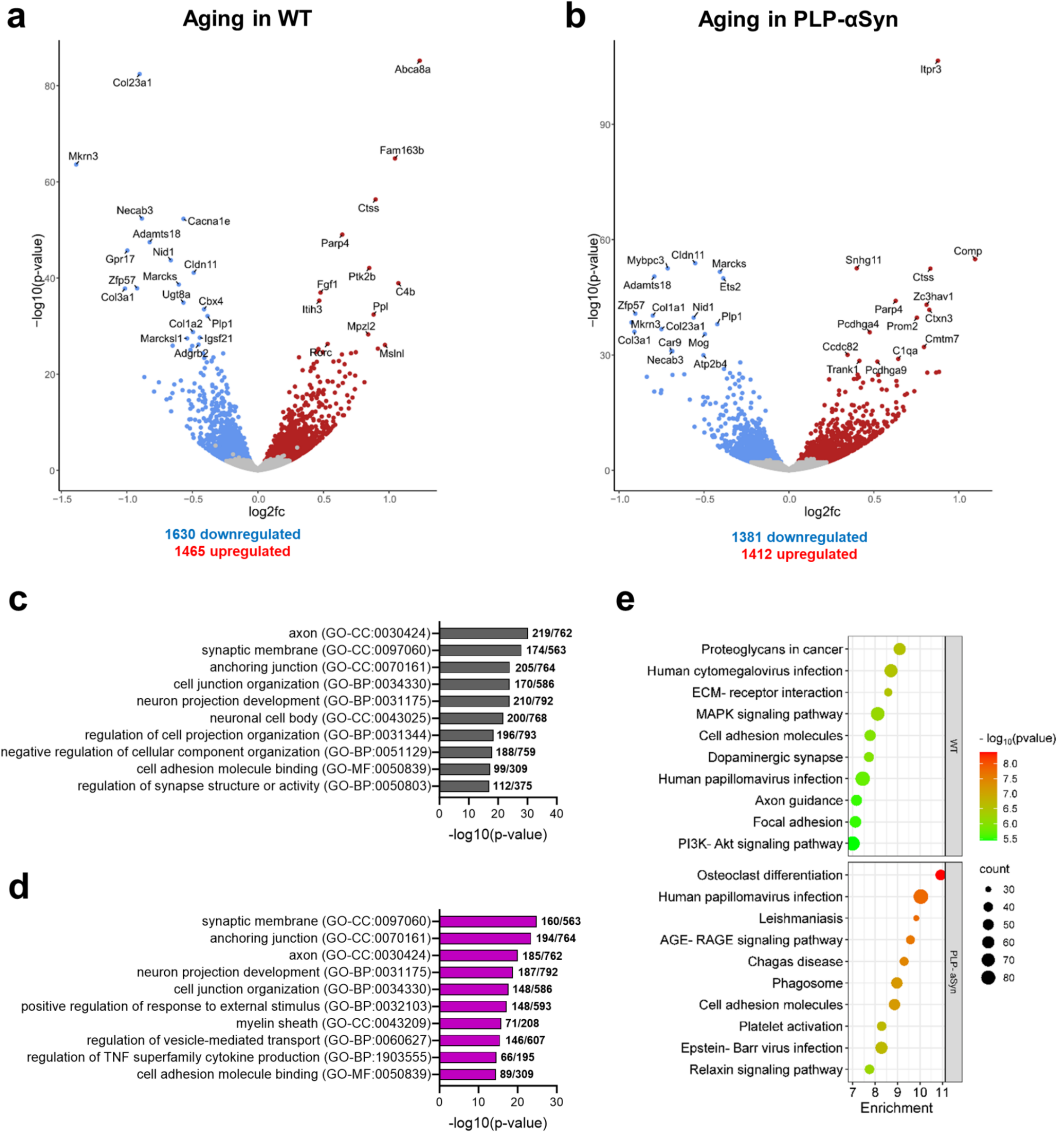
RNA-Seq analysis of cerebellar changes through aging in WT and MSA mice. **A**, Volcano plot showing the DEGs in the cerebellum of 12 versus two-month-old WT mice. Blue: downregulated; Red: upregulated; Grey: non-significant. DEGs with an adjusted p-value < 0.05 were considered significant. **B**, Volcano plot showing the DEGs in the cerebellum of 12 versus two-month-old MSA mice (TG). **C**, Top 10 GO clusters enriched through aging in cerebellum of WT mice. **D**, Top 10 GO clusters enriched through aging in cerebellum of MSA mice. **E**, Top 10 KEGG pathways enriched through aging in WT and TG mice

In order to evaluate whether the transcriptional changes observed in the cerebellum of MSA mice constitute an acceleration or aggravation of age-related transcriptional changes, DESeq2 analysis of cerebellar tissue from 12 versus 2-month-old WT control mice was performed. Our DESeq2 analysis demonstrated the dysregulation of an important number of genes in 12-month-old WT mice compared to young animals (Fig. 4a and Supplementary file). An overview of the top 30 DEG suggests alterations in several key processes including lipid metabolism (*Abca8a*, *Ugt8a*) and myelination (*Abca8a*, *Cldn11*, *Gpr17*, *Mpzl2*, *Plp1*, *Ugt8a*). Dysregulation of genes associated with extracellular matrix remodelling, tissue integrity, cellular adhesion and migration (*Adamts18*, *Cldn11*, *Col3a1*, *Col1a2*, *Col23a1*, *Itih3*, *Mslnl*, *Nid1*, *Ppl*) could be associated with alterations in ECM composition and the cellular architecture of the cerebellum. The dysregulation of genes involved in calcium signalling (*Cacna1e*, *Necab3*) may lead to disruptions in neuronal excitability and signalling. DESeq2 analysis also showed dysregulation of genes involved in cell signalling (*Cacna1e*, *Gpr17*, *Marcks*,* Ptk2b*), synaptic function (*Fam163b*, *Fgf1*, *Igsf21*, *Mkrn3*, *Necab3*, *Ptk2b*), epigenetic regulation (*Zfp57*), angiogenesis (*Adgrb2*), immune modulation and inflammation (*C4b*, *Marcks*, *Rorc*) and stress response mechanisms (*Cbx4*, *Ctss*, *Fgf1*, *Marcksl1*, *Parp4*). These changes in multiple pathways suggest that normal aging in the cerebellum leads to progressive dysfunction across neuronal, glial, and immune systems, that might set the stage for the development of neurodegenerative processes.

GO analysis of DEGs in 12-month-old versus two-month-old WT mice revealed significant enrichment of terms associated with neuronal connectivity, synaptic integrity, and cellular organization (Fig. 4c). Enriched terms “axon”, “synaptic membrane”, “neuron projection development” and “regulation of synapse structure or activity” suggest age-related alterations in synaptic function, axonal structure, and neuronal plasticity with aging. Furthermore, pathways related to cell adhesion, including “anchoring junction” “cell junction organization” and “cell adhesion molecule binding” may indicate age-associated changes in cellular interactions and tissue stability. Additionally, the enrichment of “neuronal cell body” and “negative regulation of cellular component organization” points to disruptions in cellular architecture and maintenance. KEGG analysis of those DEGs revealed significant enrichment in pathways related to cellular signalling, adhesion, and synaptic function, which are key biological processes in the aging brain (Fig. 4e). Pathways such as “Proteoglycans in cancer”, “ECM-receptor interaction” and “Focal adhesion” indicate changes in extracellular matrix remodelling and cellular adhesion, potentially contributing to cellular dysfunction during aging. Additionally, pathways like “MAPK signaling”, “PI3K-Akt signaling” and “Axon guidance” suggest disruptions in cellular signalling, stress responses, and neuronal connectivity. The “Dopaminergic synapse” and “Human cytomegalovirus infection” pathways point to impairments in synaptic function and immune responses. These findings highlight the complex interplay between cellular stress, inflammation, and synaptic function during normal aging in WT mice. Notably, processes typically observed during aging in WT mice, such as synaptic and structural changes, were already present in young MSA mice compared to controls, indicating an acceleration of aging mechanisms in PLP-αSyn mice triggered by the oligodendroglial α-synucleinopathy.

In MSA mice, DESeq2 analysis of aging and disease progression was also associated with important transcriptional dysregulation (Fig. 4b and Supplementary file). Many of the top dysregulated genes were shared with the ones observed in WT mice, and involved in synaptic function and neuronal connectivity (*Atp2b4*, *Ctxn3*, *Necab3*, *Pcdhga4*, *Pcdhga9*), extracellular matrix remodelling and cellular adhesion (*Adamts18*, *Ccdc82*, *Col1a1*, *Col3a1*, *Col23a1*, *Comp*, *Nid1*), immune responses, cellular stress and regulatory mechanisms (*C1qa*,*Ctss*, *Ets2*, *Marcks*, *Parp4*, *Prom2*, *Trank1*, *Zc3hav1*,), epigenetic regulation (*Zfp57*), lipid metabolism and myelination (*Cldn11*, *Cmtm7*, *Itpr3*, *Mog*, *Plp1*). GO analysis of DEGs between 12-month-old and two-month-old MSA mice revealed significant alterations in processes critical to neuronal and immune function (Fig. 4d). The enrichment of “synaptic membrane”, “axon”, and “neuron projection development” suggests disruptions in synaptic function and neuronal connectivity, essential for cerebellar communication and plasticity. Terms including “anchoring junction” and “cell junction organization” highlight compromised structural integrity and cellular adhesion, while enrichment in “myelin sheath” indicates potential impairments in myelination and oligodendrocyte function. Immune dysregulation and inflammation seem to be prominent in aged MSA mice, with a significant enrichment of terms like “regulation of TNF superfamily cytokine production” and “positive regulation of response to external stimulus”. The KEGG pathway analysis of DEGs between old and young MSA mice also highlighted a significant enrichment in pathways related to inflammation, immune responses, and cellular signalling (Fig. 4e). In general, the enrichment in KEGG pathways was considerably more significant than in WT mice. Notably, pathways such as “Osteoclast differentiation”, “AGE-RAGE signaling”, and “Phagosome” suggest important immune dysregulation and cellular stress responses. The enrichment of “Leishmaniasis”, “Chagas disease”, and “Epstein-Barr virus infection” pathways further supports immune dysfunction, potentially exacerbating inflammation. “Cell adhesion molecules” and “Platelet activation” pathways might indicate alterations in cellular adhesion and vascular function, while the “Relaxin signaling” pathway suggests changes in tissue remodelling and extracellular matrix dynamics. Altogether, aging in the cerebellum of MSA mice is associated with progressive synaptic, structural, and immune dysfunction, driven in part by heightened neuroinflammation and stress-related disruptions.

**Fig. 5:**
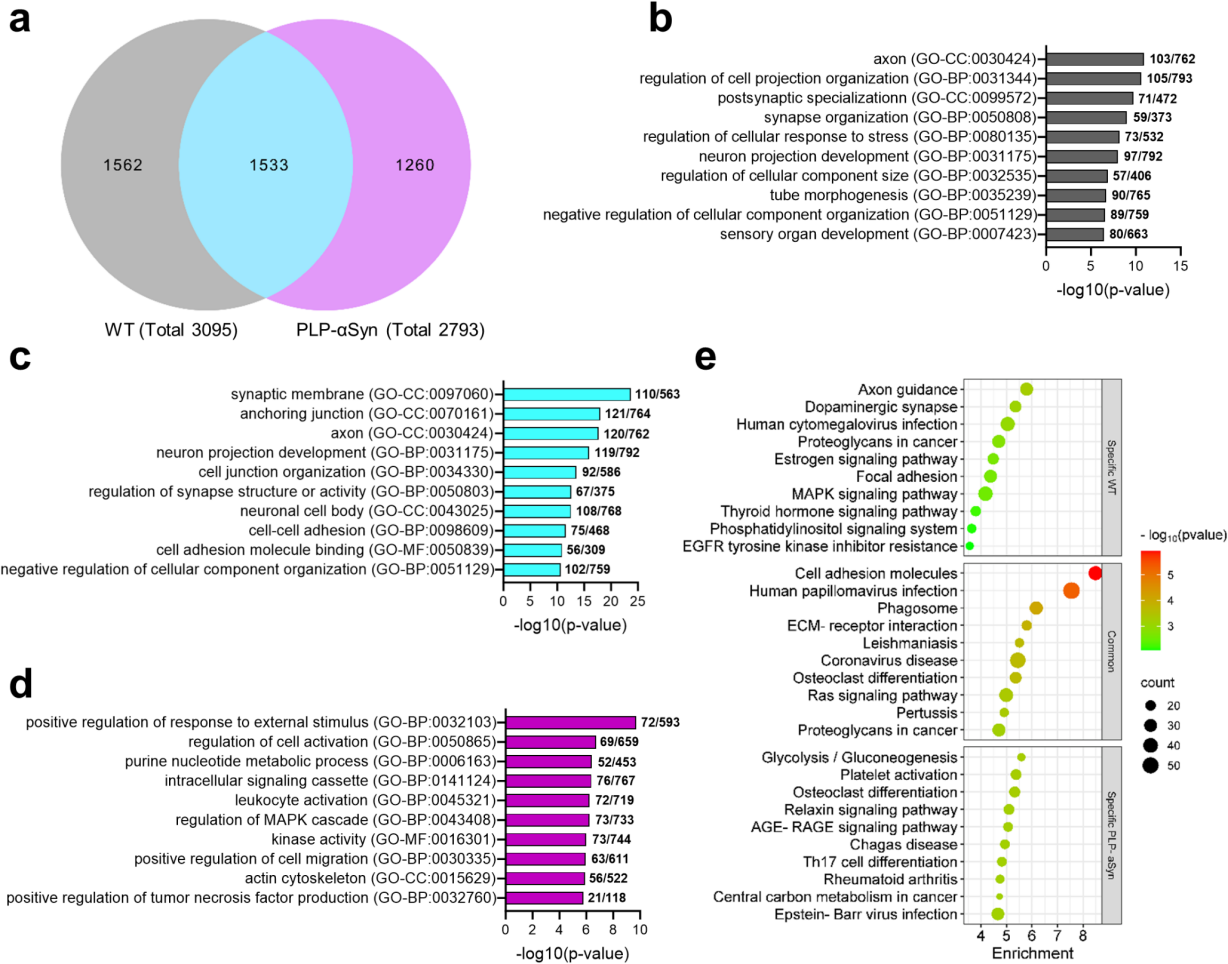
Shared and specific DEGs through aging in WT and MSA mice. **A**, Venn Diagram indicating the overlap between aging-related DEGs in WT and PLP-αSyn mice. **B**, Top 10 GO clusters from genes specifically dysregulated through aging in control mice. **C**, Top 10 GO clusters from genes dysregulated through aging in both WT and MSA mice. **D**, Top 10 GO clusters from genes specifically dysregulated through aging in PLP-αSyn mice. **E**, KEGG pathways from DEGs specifically dysregulated through in WT, DEGs shared between WT and transgenic (TG), or DEGs specifically dysregulated in MSA mice

Finally, a comparison of aging-related DEGs in WT and PLP-αSyn mice revealed the presence of both a substantial overlap and distinct sets of DEGs (Fig. 5a). GO analysis of those DEGs showed shared but also specific molecular processes, highlighting differences in cerebellar aging dynamics between WT and MSA mice. Shared processes, such as impairments in synaptic organization (e.g., “synaptic membrane”, “synapse organization”) and neuronal connectivity (“axon”, “neuron projection development”), point to common age-related disruptions in neuronal structure and function (Fig. 5c). Additionally, deficits in cell adhesion and cellular organization (“cell junction organization”, “cell-cell adhesion”) were enriched in both groups, suggesting a fundamental loss of structural integrity during aging (Fig. 5c). WT-specific changes included axonal specialization (“postsynaptic specialization”), stress regulation, and developmental processes (“tube morphogenesis”, “sensory organ development”), reflecting a potential focus on maintaining neuronal and structural stability (Fig. 5b). In contrast, MSA-specific processes included pronounced inflammatory responses (“positive regulation of response to external stimulus”, “leukocyte activation”, “positive regulation of TNF production”), intracellular signalling disruptions (e.g., “regulation of MAPK cascade”, “kinase activity”), and altered cellular metabolism (“purine nucleotide metabolic process”) (Fig. 5d). KEGG analysis of aging-related DEGs also showed distinct and overlapping pathways between WT and MSA mice (Fig. 5e). In control mice, specific aging-related DEGs lead to an enrichment in pathways such as “Axon guidance”, “Dopaminergic synapse”, and “MAPK signaling”, highlighting synaptic dysfunction, neuronal connectivity deficits, and intracellular signalling changes, consistent with our previous GO analysis of those DEGs. Shared pathways between WT and MSA mice, including “Cell adhesion molecules”, “Phagosome”, and “ECM-receptor interaction”, aligned with common GO terms related to cell adhesion, junction organization, and membrane remodelling, suggesting that structural reorganization and immune-related processes are intrinsic to aging in both conditions. Whereas, MSA-specific pathways, such as “AGE-RAGE signaling”, “Th17 cell differentiation” and “Glycolysis/Gluconeogenesis”, indicated accelerated or exacerbated inflammatory responses, immune activation, and metabolic dysregulation, corroborating MSA-specific GO terms related to leukocyte activation, cytokine regulation, and energy metabolism. These findings suggest that while aging in both WT and MSA mice is associated with synaptic and structural deficits, disease progression in the cerebellum of MSA mice is marked by accelerated inflammatory activation, metabolic disturbances and dysregulation of signalling pathways, and highlight the dual impact of MSA-like pathology, which not only accelerates age-related processes but also drives distinct pathological pathways in the cerebellum.

## Discussion

Our analysis of differential gene expression between the cerebellum of PLP-αSyn and control mice demonstrates significant alterations in several key biological processes that might provide novel insights into the pathophysiology of the cerebellar component of MSA. These findings highlight the complex interplay between aging, neurodegeneration, and disease-specific mechanisms in the cerebellum of MSA mice, a region critical for motor control and coordination.

Cerebellar pathology in MSA is characterized by progressive degeneration of neurons, particularly in the olivopontocerebellar region, with extensive loss of Purkinje cells and glial dysfunction and/or activation (Ciolli et al. 2014; Rusholt et al. 2020; Stefanova and Wenning 2023). Studies have also shown altered cerebellar volume and functional connectivity in MSA brains, which correlate with motor deficits in patients (Rusholt et al. 2020; Yang et al. 2019). Previous studies demonstrated neuronal loss in the cerebellum of PLP-αSyn mice in the presence of a second deleterious stimulus like oxidative stress or proteasome inhibition, while the same stress stimuli had no neurodegenerative effects in the cerebellum of WT mice (Stefanova et al. 2005, 2012).Our study is the first to report substantial dysregulation of genes involved in synaptic function, cellular signalling, and cellular adhesion in the cerebellum of MSA mice triggered by the oligodendroglial α-synucleinopathy. Specifically, genes related to axonal guidance, synaptic function, and postsynaptic structures were differentially expressed in MSA cerebellum compared to controls, highlighting disturbances in neuronal connectivity that could contribute to cerebellar dysfunction. In addition to synaptic alterations, our results also indicate a significant dysregulation of genes involved in inflammation, immune responses and microglial activation. This suggests that inflammation may play a critical role in MSA-like disease progression in this brain area. Chronic neuroinflammation, particularly microglial activation and cytokine dysregulation, has been linked to the progression of neurodegenerative diseases, including MSA (Gao et al. 2023; Martirosyan et al. 2024; Refolo and Stefanova 2019). Our results align with this, showing an accelerated inflammatory response in MSA mice, which could contribute to the exacerbation of cerebellar pathology.

The early dysregulation of cellular signalling pathways in the MSA cerebellum is another significant finding. In MSA mice, we observed an upregulation of pathways associated with stress responses, such as MAPK signalling, PI3K-Akt signalling, and cellular stress regulation, which are common features of neurodegenerative diseases (Goyal et al. 2023; Kim and Choi 2010; Martirosyan et al. 2024). These pathways are involved in cellular survival, apoptosis, and response to external stressors, which could suggest an attempt by cerebellar neurons to cope with the pathological changes induced by α-syn accumulation. Moreover, these alterations occurred in parallel with changes in the ECM and cell adhesion, as observed by the differential expression of genes linked to ECM remodelling and cellular communication. Such remodelling may affect synaptic integrity and contribute to motor deficits in MSA (Rosh et al. 2024; Soles et al. 2023; Yang et al. 2023).

Another interesting finding of our study is the observation that many of the processes typically associated with normal aging in WT mice were already apparent in two-month-old MSA mice, including synaptic and structural changes. These results suggest that oligodendroglial α-synucleinopathy may accelerate the normal aging process, particularly in the cerebellum, which is consistent with the hypothesis that neurodegenerative diseases can hasten age-related mechanisms (Lopez-Otin et al. 2013; Mattson and Arumugam 2018). In fact, different studies have demonstrated that age-related changes in synaptic plasticity and structure are exacerbated in the presence of α-syn aggregates, the pathological hallmark of MSA (Kulkarni et al. 2022; Sharma and Burre 2023). Therefore, oligodendroglial α-synucleinopathy may accelerate the aging of cerebellar neurons, promoting an earlier onset of synaptic dysfunction and motor impairment. Furthermore, KEGG analysis of DEGs in MSA mice revealed a complex network of pathways related to immune activation, cell adhesion, and stress responses. Notably, pathways such as “Osteoclast differentiation” and “Human papillomavirus infection” were specifically dysregulated in MSA, further supporting the idea that immune and viral response pathways may also play a role in the pathogenesis of MSA (Corbin-Stein et al. 2024; D’Sa et al. 2024; Williams et al. 2020). Altogether, these results suggest that the disease not only accelerates normal aging processes but also induces distinct pathological changes in the cerebellum, which may contribute to the characteristic clinical features of MSA.

There are important limitations in our study that must be considered. We employed a transgenic mouse model of MSA that overexpresses human wild type α-syn within oligodendrocytes. This model effectively mimics essential pathological features of MSA, such as α-syn aggregation in oligodendrocytes, SND, and motor and non-motor symptoms. Furthermore, the model is strongly supported by recent findings of increased SNCA mRNA in MSA oligodendroglia with inclusion pathology (Kon et al. 2024) as well as the presence of SNCA copy number variant mosaicism in MSA oligodendrocytes (Garcia-Segura et al. 2023). However, the chronic overexpression of α-syn in this model differs from the dynamic aggregation and spreading proposed to occur in human MSA, where these processes likely involve cell-to-cell propagation and complex interactions within the central nervous system (Stefanova 2023). Additionally, since our analysis used bulk cerebellar tissue, we cannot distinguish gene expression changes specific to individual cell types, potentially masking subtler changes unique to certain cell populations. Moreover, the cerebellum contains both grey and white matter, which possess distinct functions, and the inability to distinguish between these regions could also mask region-specific changes. Future studies that combine single-cell RNA sequencing and spatial transcriptomics may offer more detailed insights into the cellular and regional contributions to the observed pathological changes in MSA-like disease progression.

The fact that MSA is a rare neurodegenerative disorder, and that molecular evaluation of brain samples is only possible postmortem at end stages, constitutes a significant limitation for deciphering the biological mechanisms underlying MSA disease progression. For this reason, the use of animal models, such as the one employed in this study, provides a valuable tool to identify early pathological events and processes that might play a central role in MSA pathogenesis. The results presented here provide, for the first time, novel insights into the molecular changes associated with oligodendroglial α-synucleinopathy in the cerebellum of MSA mice during the early stages of disease progression. Our findings reveal an acceleration of aging mechanisms and the involvement of inflammation, synaptic dysfunction and dysregulation of key signalling pathways. These pathological alterations occur at early disease stages, worsen with aging, and precede neuronal loss. Therefore, the molecular mechanisms identified in MSA mice may play a crucial role in cerebellar pathogenesis in human MSA and could have translational implications. Given that synaptic dysregulation and neuroinflammation constitute central pathological events in disease progression triggered by oligodendroglial α-synucleinopathy, targeting these processes may offer relevant therapeutic benefits. In this regard, anti-inflammatory strategies targeting microglial activation and neuroinflammation, or inhibiting specific signalling pathways involved in synaptic plasticity (e.g., MAPK or PI3K-Akt), could offer a potential approach to slow disease progression, improve motor function, or alleviate symptoms in future clinical trials for this devastating disease.

## Electronic supplementary material

Below is the link to the electronic supplementary material.

Supplementary Material 1
